# Supplementary material for: Metabolic co-dependence drives the evolutionarily ancient Hydra–Chlorella symbiosis
Source: eLife. 2018 May 31;7:e35122. doi: 10.7554/eLife.35122 (PMC6019070; doi:10.7554/eLife.35122)
Supplement: Supplementary file 2. — (B) Blast best hit genes of Arabidopsis thaliana in Chlorella sp. A99 genes belonging to OG0000040 and OG0000324. [file elife-35122-supp2.docx]

**Supplementary File 2**

A. Ortholog groups of Aa_trans containing protein in *Chlorella variabilis NC64A* (NC64A), *Coccomyxa subellipsoidea* C-169 (C169), *Volvox carteri* (Vc), *Micromonas pusilla* (Mp) and *Ostreococcus tauri* (Ot) and *Chlamydomonas reinhardtii* (Cr).

| **Ortholog Group ID** | **A99** | **NC64A** | **c169** | **Cr** | **Vc** | **Mp** | **Ot** |
| --- | --- | --- | --- | --- | --- | --- | --- |
| OG0000040 | 12 | 12 | 6 | 3 | 1 | 0 | 0 |
| OG0000324 | 6 | 7 | 1 | 2 | 1 | 0 | 0 |
| OG0001336 | 2 | 1 | 1 | 1 | 1 | 1 | 1 |
| OG0004053 | 1 | 2 | 2 | 1 | 0 | 0 | 0 |
| OG0006517 | 1 | 1 | 0 | 0 | 0 | 0 | 0 |
| OG0001069 | 1 | 2 | 3 | 1 | 1 | 1 | 1 |
| OG0000830 | 1 | 4 | 1 | 2 | 1 | 2 | 1 |
| OG0002190 | 1 | 1 | 1 | 1 | 1 | 1 | 1 |
| OG0011340 | 1 | 0 | 0 | 0 | 0 | 0 | 0 |
| OG0004863 | 1 | 5 | 0 | 0 | 0 | 0 | 0 |
| OG0000468 | 2 | 2 | 2 | 2 | 2 | 2 | 2 |
| OG0003354 | 2 | 1 | 1 | 1 | 1 | 1 | 1 |
| OG0003801 | 1 | 1 | 0 | 1 | 1 | 1 | 1 |

B. Blast best hit genes of *Arabidopsis thaliana* in *Chlorella* sp. A99 genes belonging to OG0000040 and OG0000324

| **OG0000040** | **Best Hit gene ID** | **Best Hit gene of Arabidopsis thaliana** | **e-value** |
| --- | --- | --- | --- |
| scaffold1.g5447.t1 | NP_196484.1 | amino acid permease 2 | 4E-62 |
| scaffold1.g5579.t1 | NP_196484.1 | amino acid permease 2 | 7E-32 |
| scaffold12.g8277.t1 | NP_196484.1 | amino acid permease 2 | 1E-34 |
| scaffold13.g380.t1 | NP_196484.1 | amino acid permease 2 | 6E-12 |
| scaffold14.g1284.t1 | NP_196484.1 | amino acid permease 2 | 2E-33 |
| scaffold2.g7119.t1 | NP_001318716.1 | lysine histidine transporter 1 | 9E-73 |
| scaffold2.g7251.t1 | NP_196484.1 | amino acid permease 2 | 3E-59 |
| scaffold21.g2221.t1 | NP_175076.2 | amino acid permease 5 | 9E-60 |
| scaffold40.g5168.t1 | NP_196484.1 | amino acid permease 2 | 5E-38 |
| scaffold5.g864.t1 | NP_196484.1 | amino acid permease 2 | 2E-60 |
| scaffold6.g2644.t1 | NP_196484.1 | amino acid permease 2 | 2E-58 |
| scaffold6.g2815.t1 | NP_186825.2 | Transmembrane amino acid transporter family protein | 3E-43 |
| **OG0000324** | **Best Hit gene ID** | **Best Hit gene of Arabidopsis thaliana** | **e-value** |
| scaffold10.g2481.t1 | NP_001330273.1 | Transmembrane amino acid transporter family protein | 7E-06 |
| scaffold11.g3916.t1* | NP_172258.1  NP_565239.1 | Histone superfamily protein  Transmembrane amino acid transporter family protein | 1E-47  7E-40 |
| scaffold15.g4364.t1 | NP_566854.1 | Transmembrane amino acid transporter family protein | 8E-31 |
| scaffold2.g7197.t1 | NP_566854.1 | Transmembrane amino acid transporter family protein | 2E-24 |
| scaffold21.g2185.t1 | NP_565239.1 | Transmembrane amino acid transporter family protein | 5E-14 |
| scaffold3.g6325.t1 | NP_566854.1 | Transmembrane amino acid transporter family protein | 6E-34 |

* This sequence consists from a region similar to NP_172258.1 and a region similar to NP_565239.1.
