# Supplementary material for: Metabolic co-dependence drives the evolutionarily ancient Hydra–Chlorella symbiosis
Source: eLife. 2018 May 31;7:e35122. doi: 10.7554/eLife.35122 (PMC6019070; doi:10.7554/eLife.35122)
Supplement: Supplementary file 4. [file elife-35122-supp4.docx]

**Supplementary File 4**

Sequence ID of nitrogen assimilation genes in *Symbiodinium*.

|  | ***S. minutum*** | ***S. kawagutii*** | ***S. microadriaticum*** |
| --- | --- | --- | --- |
| **nitrate/nitrite transporter** | symbB.v1.2.016992.t1 | Skav225355, Skav219905, Skav225351, Skav203483, Skav209212, Skav201769, Skav225357, Skav201780, Skav228348, Skav225353, Skav207686, Skav207688, Skav209211, Skav209986, Skav219909, Skav219911 | Smic4625, Smic4635, Smic4642, Smic4659, Smic8092, Smic16438, Smic19954, Smic19956, Smic19959, Smic39394, Smic39591, Smic42197, Smic46994 |
| **nitrate reductase** | symbB.v1.2.040297.t1, symbB.v1.2.039611.t1, symbB.v1.2.036677.t1 | Skav200060 Skav201768 Skav201772 Skav201782 Skav203995 Skav204326 Skav207684 Skav208446 Skav214906 | Smic18218, Smic29038, Smic32352 |
| **nitrite reductase** | symbB.v1.2.039610.t1 symbB.v1.2.040143.t1 symbB.v1.2.036678.t1 | Skav201776, Skav201775, Skav201565, Skav201710, Skav225196, Skav210721, Skav210720, Skav209209 | Smic13334, Smic21292, Smic36135, Smic36136, Smic36137, Smic46498 |
| **glutamine synthetase** | symbB.v1.2.034154.t1, symbB.v1.2.034155.t1, symbB.v1.2.021192.t1, symbB.v1.2.021193.t1, symbB.v1.2.024504.t1, symbB.v1.2.000900.t1, symbB.v1.2.019913.t1, symbB.v1.2.034154.t1, symbB.v1.2.034155.t1, symbB.v1.2.021192.t1, symbB.v1.2.021193.t1, symbB.v1.2.024504.t1, symbB.v1.2.000900.t1, symbB.v1.2.019913.t1, symbB.v1.2.034154.t1, symbB.v1.2.034155.t1, symbB.v1.2.021192.t1, symbB.v1.2.021193.t1, symbB.v1.2.024504.t1, symbB.v1.2.000900.t1, symbB.v1.2.019913.t1, symbB.v1.2.034154.t1, symbB.v1.2.034155.t1, symbB.v1.2.021192.t1, symbB.v1.2.021193.t1, symbB.v1.2.024504.t1, symbB.v1.2.000900.t1, symbB.v1.2.019913.t1 | Skav214369, Skav229194, Skav210471, Skav217577, Skav207474, Skav200954, Skav211054, Skav212873, Skav200952, Skav211053, Skav221126, Skav202590, Skav218541 | Smic2606, Smic11656, Smic15228, Smic17455, Smic21035, Smic23174, Smic38039, Smic46769 |
| **glutamate synthase (ferredoxin)** | symbB.v1.2.020534.t | Skav221682 | Smic28419 |
| **glutamate synthase (NADPH/NADH)** | symbB.v1.2.014989.t1, symbB.v1.2.017142.t1, symbB.v1.2.017146.t1, symbB.v1.2.014989.t1, symbB.v1.2.017142.t1, symbB.v1.2.017146.t1 | Skav230375, Skav230378, Skav229867, Skav230379, Skav229868, Skav230377, Skav229865, Skav230376, Skav233007 | Smic9540, Smic27011 |

Sequences were obtained from the database of OIST Marine Genomics in *S. minutum, Symbiodinium kawagutti* Genome and Dinoflagellate Resources in *S. kawagutii* and Reefgenomics in *S. microadriaticum.* The annotation of the genes in *S. kawagutii* was based on Lin et al., (2015).
