# Supplementary material for: Metabolic co-dependence drives the evolutionarily ancient Hydra–Chlorella symbiosis
Source: eLife. 2018 May 31;7:e35122. doi: 10.7554/eLife.35122 (PMC6019070; doi:10.7554/eLife.35122)
Supplement: Supplementary file 5. [file elife-35122-supp5.docx]

**Supplementary File 5**

Sequence ID of nitrogen assimilation genes in *Chlorella variabilis NC64A* (NC64A), *Coccomyxa subellipsoidea* C-169 (C169), *Volvox carteri* (Vc), *Micromonas pusilla* (Mp) and *Ostreococcus tauri* (Ot) and *Chlamydomonas reinhardtii* (Cr).

| **A99** | **NC64A** | **c169** | **Cr** | **Vol** | **Mp** | **Ot** |
| --- | --- | --- | --- | --- | --- | --- |
| **GOGAT (glutamate synthase)*** | | | | | | |
| scaffold18.g1969.t1 | 33619 | 22625 | Cre12.g514050 | Vocar.0006s0290 | 57115 | 29431 |
|  | 142154 | 53183 | Cre13.g592200 | Vocar.0064s0005 | 70244 |  |
| **GS (Glutamine synthetase)*** | | | | | | |
| scaffold6.g2861.t1 | 56005 | 23194 | Cre02.g113200 | Vocar.0001s1591 | 4228 | 15060 |
| scaffold7.g3389.t1 | 143431 | 23517 | Cre03.g207250 | Vocar.0011s0254 |  |  |
|  |  | 30043 | Cre12.g530600 | Vocar.0028s0089 |  |  |
|  |  | 31742 | Cre12.g530650 | Vocar.0028s0090 |  |  |
| **AMT (ammonium transporter, Rh)** | | | | | | |
| na | 21763 | 65570 | Cre06.g284100 | na | na | na |
|  |  | 65572 | Cre06.g284150 |  |  |  |
| **AMT (ammonium transporter, Amt family)** | | | | | | |
| scaffold2.g7405.t1 | 36096 | 47532 | Cre02.g111050 | Vocar.0001s1695 | 29536 | 29863 |
| scaffold2.g7406.t1 | 56592 | 52218 | Cre03.g159254 | Vocar.0008s0224 | 45964 | 18135 |
| scaffold3.g6262.t1 | 58614 |  | Cre06.g293051 | Vocar.0019s0251 | 48406 | 25714 |
|  | 136742 |  | Cre07.g355650 | Vocar.0022s0058 | 50351 | 29181 |
|  | 141357 |  | Cre09.g400750 | Vocar.0028s0082 | 59331 |  |
|  |  |  | Cre12.g531000 | Vocar.0049s0040 |  |  |
|  |  |  | Cre13.g569850 | Vocar.0051s0018 |  |  |
|  |  |  | Cre14.g629920 | Vocar.0054s0046 |  |  |
|  |  |  |  | Vocar.0063s0027 |  |  |
|  |  |  |  | Vocar.0069s0013 |  |  |
| **NRT2 (high affnity nitrate/nitrite transporter)*** | | | | | | |
| na | 26630 | 28993 | Cre02.g110800 | Vocar.0008s0137 | 49583 | 24168 |
|  | 138674 |  | Cre03.g150101 | Vocar.0008s0138 |  |  |
|  |  |  | Cre03.g150151 | Vocar.0008s0200 |  |  |
|  |  |  | Cre09.g396000 |  |  |  |
|  |  |  | Cre09.g410800 |  |  |  |
|  |  |  | Cre09.g410850 |  |  |  |
| **NRT1 (nitrate/peptide transporter family, low affinity nitrate transporter)*** | | | | | | |
| scaffold14.g1044.t1 | 23105 | 19259 | Cre04.g224700 | Vocar.0049s0020 | na | 2706 |
|  | 144528 | 48785 |  |  |  |  |
|  |  |  |  |  |  |  |
| **NAR1 (formate-nitrite transporter family)*** | | | | | | |
| scaffold12.g8267.t1 | 34412 | 20872 | Cre01.g012050 | Vocar.0007s0061 | 70731 | 19784 |
| scaffold18.g1993.t1 | 25301 | 46302 | Cre04.g217915 | Vocar.0008s0135 |  |  |
| scaffold4.g4586.t1 | 53335 |  | Cre06.g309000 | Vocar.0011s0110 |  |  |
| scaffold6.g2788.t1 |  |  | Cre07.g335600 | Vocar.0030s0055 |  |  |
|  |  |  | Cre12.g541200 | Vocar.0046s0010 |  |  |
|  |  |  | Cre12.g541250 |  |  |  |
| **NAR2 (nitrate high-affinity transporter accessory)*** | | | | | | |
| na | 138676 | 47957 | Cre09.g410900 | Vocar.0008s0139 | 70904 | 24167 |
| **NiR (ferredoxin-nitrite reductase)*** | | | | | | |
| na | 26644 | 29833 | Cre09.g410750 | Vocar.0008s0136 | 70828 | 26396 |
| **NR (nitrate reductase (NAD(P)H ))*** | | | | | | |
| scaffold14.g1111.t1 | 56304 | 37154 | Cre09.g410950 | Vocar.0008s0140 | 39565 | 19576 |
|  |  | 29226 |  |  | 57689 |  |

Sequences were obtained from the database of JGI genome portal for *Chlorella variabilis NC64A* (NC64A), *Coccomyxa subellipsoidea* C-169 (C169), *Volvox carteri* (Vc), *Micromonas pusilla* (Mp) and *Ostreococcus tauri* (Ot) and of Phytozome for *Chlamydomonas reinhardtii* (Cr).

* The number of genes in NC64A, C169, Vc, Cr, Mp, Ot were based on Sanz-Luque et al. (2015)
