# Supplementary material for: Metabolic co-dependence drives the evolutionarily ancient Hydra–Chlorella symbiosis
Source: eLife. 2018 May 31;7:e35122. doi: 10.7554/eLife.35122 (PMC6019070; doi:10.7554/eLife.35122)
Supplement: Supplementary file 6. [file elife-35122-supp6.docx]

**Supplementary File 6**

Primers used in this study

A. Primer sequences for quantitative real time RT-PCR

| **Gene ID** | **Forward Primers** | **Reverse Primers** |
| --- | --- | --- |
| 5168 | ACTTTTCGGATATCAAACCCATTC | AATTGAACCTATTCCTCGAACGTC |
| 6508 | GCATCAAATGCGTCCAAATAAC | TTACCGAATATTCAGGCCTTTCTC |
| rc_2417 | CTTATTGCTCATGACCGTAAAGATG | TCGATTTTCACCCTTGATGG |
| 24563 | TGCGCCTTAGTTATATCTCCTCTC | TCTCTTTCTTGTGTTGTTTCTTTCC |
| rc_9398 | GATGTTTGTAGAACACGTTGGATTG | TTCAAGACAGGAGACCACAGG |
| 11411 | TCTTGCTCATGCAACACTGG | CGGTTTACTGCCAATCACATAC |
| 26108 | AATTCCTGTCCGACTGATTTCC | CCAAATCGACCCTTACTTGTTTG |
| rc_10789 | TTGCAAGAATATCTGCTGCTAAG | AGAAATCAACGGAGATCGTGTAG |
| rc_12826 | TTTATTCAAGCAATGGGCAATC | CGTTGCGTTTGTCCCTTTC |
| rc_8898 | TTAAGCATCAACGAAATATCCACTC | ACTTGTTTTGTTGCAAGTGTAGAGC |
| FV81RT001CSTY | TTAGAAATGCATGGTGTTGTTGG | CGGGTCTGTCAAGCATAAGAAG |
| RSASM_17752 | AGAATTGCTTGGGGTGTTCC | GCATATCCACGAATGAGACAAAG |
| rc_13579 | ACGGAGGTTTGGGGAAATAG | TTTGGTCTTAGGAGTGCTCGTC |
| 27417 | TGTACCTGTCCATGGAATTAAAGC | TACCTTGTCCGAATAGCAGCTC |
| rc_26218 | TTAAACTTCGAAGCTGGAAATGG | TTAGCGAAGACTTTGTCGTATGG |
| 1046 | GTGGGTTGCTCGTTATCTACTTG | CACCAGGGATGGGTTTAGG |
| rc_12891 | GTCGGTATGGGAGGTGGAG | CCCAATATACCGCCGACAG |
| NPNHRC_26859 | TGATGAACAAAAGAGCCGTATCTC | GCACGAACCGATACGTCAAC |
| RC_FVQRUGK01AXSJ | TCCCTTATGCACAGGTACGG | GGATCAATAACTGGTGGCACTG |
| rc_14793 | CACCCTTGGGCTGGTAAG | GGGATCTATGGGCAAATAAGG |
| FV81RT002HT2FL | CCAGCAAAAGCCCTTGATTAC | CCTGAATTCACCCCTCCATC |
| NPNHRC_12201 | GTCGGTATGGGAGGTGGAG | CCCAATATACCGCCGACAG |
| finalASM_15403 | AATAGGTGATGCTGGAGAGAATC | AGTATATATGGCTCTCGAGAGTG |
| finalASM_344 | ATGTGAGCCATGTCCAATTGGA | CACTTCTATTGGCAGCTTTCTC |
| tubulin alpha | TTCCTTGCTCATGGAACGAC | AGCAGGGTAAACTGCAAACTCC |
| ef1-alpha | ACCAACATTGTCACCTGGGAG | GGAACAGTACCTGTTGGTCGT |

B. Primer sequences for *in situ* hybridization probes

| **Gene Name** | **Forward Primers** | **Reverse Primers** |
| --- | --- | --- |
| GS-1 | TTGCTGACCCATTCAGAGGA | CCGAACCCAAAAGACCAAAG |
| NaPi | TTGGGAACACAACTGCTGAT | AAAGTTTAGCGAAGACTTTGTCG |
| Spot14 | GAGAAATTGATTAAGCAAGTAAGAG | GGTCAATTGCTCGGTTTC |

C. Primer sequences for PCR amplification of nitrogen assimilation genes in green algae

| **Gene** | **Forward Primers** | **Reverse Primers** |
| --- | --- | --- |
| NRT2 | YCAGTTCTGGTSCKSBRYSMTGTTC | CCCACATGGGRAASYRRATG |
| NiR | ACATCACCACVCGCGCCAACATC | TYGWRKCCMACGTCGTTGATGTG |
| NR | CTGGTGGTACMRSCCSGASTT | SAKCATSCCMATSASRTTCC |

D. Primer sequences for PCR amplification of 18S ribosomal DNA gene in green algae

| **Forward Primers** | **Reverse Primers** |
| --- | --- |
| GGAATAACACGATAGGACTCTGG | GACGGGCGGTGTGTACAAAG |
