# Supplementary material for: Metabolic co-dependence drives the evolutionarily ancient Hydra–Chlorella symbiosis
Source: eLife. 2018 May 31;7:e35122. doi: 10.7554/eLife.35122 (PMC6019070; doi:10.7554/eLife.35122)
Supplement: Supplementary file 7. [file elife-35122-supp7.docx]

**Supplementary File 7**

Composition of modified Bold’s Basal Medium for 1 liter (pH. 7)

| KH_2_PO_4_ | 175 mg |
| --- | --- |
| K_2_HPO_4_ | 100 mg |
| MgSO_4_ · 7H2O | 75 mg |
| CaCl_2_ · 2H_2_O | 25 mg |
| NaCl | 25 mg |
| FeSO_4_ · 7H_2_O, H_2_SO_4_ | 5.0 mg, 1ul |
| Na_2_EDTA, KOH | 50 mg, 31 mg |
| H_3_BO_3_ | 11.4 mg |
| ZnSO_4_ · 7H_2_O | 8.8 mg |
| MnCl_2_ · 7H_2_O | 1.4 mg |
| MoO_3_ | 0.7 mg |
| CuSO_4_ · 5H_2_O | 1.6 mg |
| Co(NO_3_)_2_ · 6H_2_O | 0.5 mg |
| glucose | 5 g |
| Vitamine B1 (Thiaminhydrochloride) | 1.2 mg |
| Vitamine B12 (Cyanocobalamin) | 0.01 mg |

Nitrogen Components

| NaNO_3_ | 250 mg |
| --- | --- |
| NH_4_Cl | 155 mg |
| Glutamine | 426 mg |
| Casamino acids | 426 mg |
